# Treatment of Undernutrition in Pregnancy Requires Adequate Food and Inflammation Control

**DOI:** 10.1016/j.advnut.2025.100479

**Published:** 2025-07-11

**Authors:** David Taylor Hendrixson, Ashleen Lee, Eliza Kleban, Kevin B Stephenson, Aminata S Koroma, Mark J Manary

**Affiliations:** 1 Department of Pediatrics, University of Washington, Seattle, WA, United States; 2 Project Peanut Butter, Freetown, Sierra Leone; 3 Department of Pediatrics, Washington University in St. Louis, St. Louis, MO, United States; 4 Department of Medicine, Washington University in St. Louis, St. Louis, MO, United States; 5 Directorate of Nutrition, Sierra Leone Ministry of Health, Freetown, Sierra Leone

**Keywords:** undernutrition, pregnancy, ready-to-use supplemental food, multiple micronutrients, fetal growth: balanced energy and protein supplements, lipid nutrient supplement, inflammation

## Abstract

Undernutrition in pregnancy remains a substantial problem worldwide, disproportionately affecting females living in low- and middle-income countries where food insecurity and limited access to high-quality nutrition exacerbate maternal and fetal health risks. Undernutrition during this critical time in the lifecycle can have adverse effects on both the pregnant female and her offspring. Despite the widespread recognition of this issue, current international guidelines provide insufficient direction on optimal nutritional management strategies. Most clinical trial evidence has yielded inconclusive results. This perspective synthesizes evidence on current management strategies in addressing macronutrient and micronutrient deficiencies in pregnancy. Additionally, we examine the critical role of inflammation in moderating the effectiveness of nutritional interventions and discuss emerging strategies that integrate infection control with nutrition to optimize maternal and neonatal outcomes. Given the limitations of existing management strategies, there is an urgent need for more comprehensive, evidence-based guidelines to improve pregnancy outcomes for undernourished females worldwide.

**Statements of Significance**

The perspective provides evidence that undernutrition in pregnancy remains an important problem globally and current management strategies remain insufficient. On the basis of these findings, the perspective urges further research and international support to identify optimal strategies to manage this serious condition.

## Introduction

Undernutrition in pregnancy, defined by anthropometry, affects nearly 10% of pregnant people worldwide [1]. Undernutrition is particularly recalcitrant among pregnant females aged 15–19 y [2], especially in low- and middle-income countries (LMICs), where rates are ≤10 times higher than in wealthier nations [3]. Approximately 24% of pregnancies are affected in sub-Saharan Africa [4], with a meta-analysis indicating that 20% of pregnant females are underweight, and over 70% of these females experience inadequate gestational weight gain [5].

Undernutrition during pregnancy not only compromises maternal health, conferring increased risk of hypertensive disorders, infections [6], and pregnancy-related mortality [7], but also has consequences for fetal and infant outcomes. Reductions in placental size and blood flow, resulting in reduced nutrient delivery, can result in fetal growth restriction and preterm birth [8]. Furthermore, maternal undernutrition is associated with long-term health risks in the offspring, including cardiovascular disease, hypertriglyceridemia, impaired glucose tolerance, and obesity in adulthood [9,10].

Food insecurity has increased since 2014, affecting nearly 12% of the global population, with a 10% higher prevalence among females compared with males [11]. Females living in rural LMIC settings have a 2.6-fold greater likelihood of undernutrition during pregnancy compared with urban females [4]. Adolescents face a greater risk due to nutrient competition and limited nutritional reserves [12]. In addition to food insecurity, inflammation compromises optimal growth and nutrient transfer by impairing intestinal absorption [13], increasing metabolic demands, and redirecting nutrients away from growth processes toward immune responses [14]. Inflammation is highly prevalent among pregnant females in LMICs [15]. Common sources of inflammation in pregnant females in LMICs include malaria [16], bacterial infections, poor oral health [17], and environmental enteropathy [13].

**FIGURE 1:**
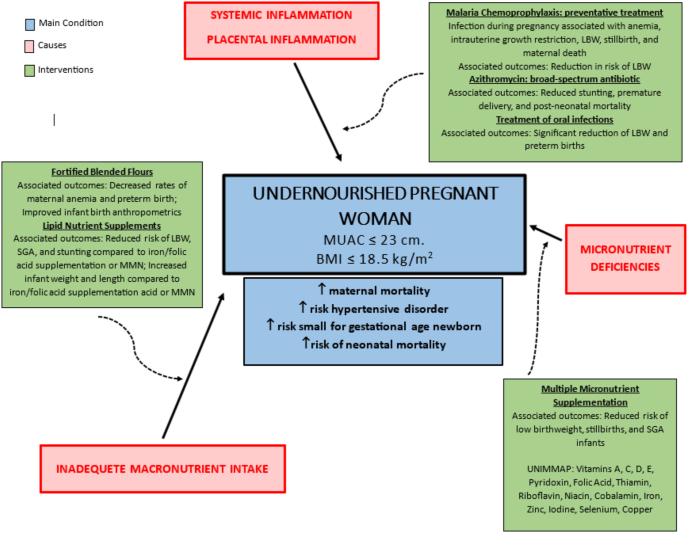
Causes and interventions of undernutrition in pregnant females. LBW, low birth weight; MMN, multiple micronutrient supplement; MUAC, mid-upper arm circumference; SGA, small-for-gestational age; UNIMMAP, United Nations International Multiple Micronutrient Antenatal Preparation.

Effective strategies to combat undernutrition must simultaneously address 3 requirements: *1*) adequate micronutrient intake, *2*) adequate macronutrient intake, and *3*) limitation of inflammation (Figure 1 and Table 1) [18–35]. These elements often coexist and exert negative synergism, underscoring the need for comprehensive interventions.

**TABLE 1:** Summary of studies of nutritional interventions for undernutrition in pregnancy

Author	Type of study	Year	Intervention studied	Location	Results
Dewidar et al. [18]	Systematic review	2023	Two-way nutritional counseling practices on maternal and infant behavioral, nutritional, and health outcomes	LMICs; 6418 records and 52 studies	Two-way interactive nutritional counseling during pregnancy may improve dietary caloric intake (MD: 81.65 calories, 95% CI: 15.37, 147.93), reduce hemorrhage [relative risk (RR): 0.63; 95% CI: 0.25, 1.54], improve protein intake (MD: 10.44 g, 95% CI: 1.83, 19.05), fat intake (MD: 3.42 g, 95% CI: −0.20, 7.04), and improve gestational weight gain within recommendations (RR: 1.84; 95% CI: 1.10, 3.09).
Ota et al. [19]	Systematic review	2015	Dietary education during pregnancy/energy and protein supplementation on maternal and infant health outcomes	LMICs; 149 reports from 65 trials	Females given nutritional education had a lower relative risk of having a preterm birth (2 trials, 449 females) [risk ratio (RR) 0.46, 95% CI: 0.21, 0.98, low-quality evidence], and LBW (1 trial, 300 females) (RR 0.04, 95% CI: 0.01, 0.14)
da Silva Lopes et al. [20]	Systematic review	2017	Nutrition-specific and nutrition-sensitive interventions to prevent LBW	LMICs; 909 records	Females who received education to boost energy and protein intake during pregnancy experienced a 96% decrease in LBW rates and a 54% decrease in PTB compared with those without such nutritional guidance.
Oh et al. [26]	Systematic review	2020	MMN; IFA vs. folic acid; MMN vs. IFA; LNS vs. MMN	LMICs; 72 studies	MMN reduced risk of low birthweight (15%), stillbirths (9%), and SGA infants (7%). LNS supplementation had no significant effect on low birthweight, perinatal mortality, miscarriage, neonatal mortality, preterm birth, or SGA infants. The evidence was of moderate to low quality,
Keats et al. [21]	Systematic review	2019	MMN supplementation	LMICs; 20 trials	MMN supplementation reduced low birthweight (LBW) (RR 0.88) and small-for-gestational-age (SGA) infants (RR 0.92), slight reduction in preterm birth (RR 0.95)
Gomes et al. [22]	Meta-analysis	2023	MMN supplementation vs. IFA	LMICs; 16 trials	MMN reduced LBW (RR 0.87), PTB (RR 0.90), and SGA (RR 0.90) compared with IFA
Vaidya et al. [23]	Follow-up of RCT	2008	Long-term effects of MMN supplementation vs. IFA	Nepal; 917 children	Birth weight was higher by 77 g (95% CI: 24, 130) in the intervention group compared with controls. Children whose mothers took MMS in pregnancy weighed 204 g more (95% CI: 27, 381), had larger head [2.4 mm (95% CI: 0.6, 4.3)], chest [3.2 mm (0.4, 6.0)], and MUACs [2.4 mm (1.1, 3.7)], as well as thicker triceps skinfolds [2.0 mm (0.0, 0.4)].
Okala et al. [24]	RCT	2019	MMN supplementation: FeFol, MMN, BEP, BEP+MMN	The Gambia; 875 females	Mean antibody titers were higher for pertussis in the BEP (by 9.4%, 3.3–15.5) and BEP + MMN groups (by 15.4%, 9.6–21.2) when compared with the FeFol group (74.9 IU/mL, 67.8–82.8)
Dhaded et al. [25]	RCT	2020	MMN supplementation: daily lipid-based micronutrient	South Asia; 972 newborns	Supplementation ≥3 mo preconception led to significant improvements in birth length (+5.3 mm) and weight (+89 g).
Lassi et al. [57]	Systematic review	2020	BEP	LMICs; 15 studies	BEP supplementation may show 40% reduction in the incidence of LBW (RR 0.60; 95% CI: 0.41, 0.86, random effect)
Janmohamed et al. [27]	RCT	2016	Fortified blended flours (corn soya blend plus)	Kampond Chhnang Province, Cambodia; 547 females	Females receiving CSB+ starting in the first trimester until delivery demonstrated doubling in the prevalence of maternal anemia which decreased to 34% as opposed to the control group with an increased prevalence of 70%; preterm birth rate was significantly lower in the treatment group (treatment: 2.1%; control: 7.1%)
Saville et al. [28]	RCT	2018	BEP of wheat-soya blended flour + super cereal	Nepal; 25,092 pregnant females	Infants in the PLA plus food group had a significantly higher birthweight compared with the control group, with an average increase of 78 g (95% CI: 13.9, 142.0). Although low birth weight (LBW) prevalence was lower in the PLA plus food group (19.1%) than the control (22.5%), this 16% decrease was not statistically significant (OR 0.74; 95% CI: 0.51, 1.08)
Ashorn et al. [46]	RCT	2015	LNS	Malawi; 1391 females	20 g sachet of SQ-LNS group had the highest mean birth weight (3000 ± 447 g) and newborn length (49.9 ± 2.1 cm), as well as the lowest prevalence of LBW (12.1%) and stunting (14.9%)
Mridha et al. [29]	RCT	2016	LNS	Bangladesh; 4011 pregnant females	20 g sachet of LNS resulted in higher birth weights (2620 ± 408 vs. 2588 ± 413 g; *P* = 0.007), WAZ (−1.48 ± 1.01 vs. −1.59 ± 1.02; *P* = 0.006), HCZ (−1.26 ± 1.08 vs. −1.34 ± 1.12; *P* = 0.028), than those in the IFA group
Adu-Afarwuah et al. [30]	RCT	2015	LNS	Ghana; 1057 females	LNS resulted in significantly higher birth weight (+85 g), WAZ (+0.19), and BMI-for-age *z*-score (+0.21) than the IFA group, with a lower risk of LBW (RR: 0.61, *P* = 0.032)
Hendrixson et al. [31]	RCT	2021	RUSF + anti-infective treatments	Sierra Leone; 1489 females	Infants of females receiving RUSF+ were 0.3 cm longer (*P =* 0.007) and weighed 70 g more (*P =* 0.005) than those receiving standard care. Weekly maternal weight gain was 40 g higher (*P =* 0.010), and neonatal deaths were reduced by 2.4% (HR 0.62, *P =* 0.026) in the RUSF+.
Adu-Afarwuah et al. [58]	RCT	2016	LNS vs. IFA	Ghana; 1320 females	SQ-LNS resulted in infants with greater mean length (79.7 cm; *P* = 0.006) and LAZ (−0.69; P = 0.009) than those receiving IFA (79.1 cm, LAZ -0.87) and MMN (79.1 cm, LAZ -0.91) groups, with a lower stunting prevalence (8.9% vs. 13.7% for IFA and 12.9% for MMN; *P* = 0.12).
Das et al. [32]	Systematic review	2018	LNS vs. IFA vs. MMN	LMICs; 8018 females	Compared with IFA, LNS resulted in slight increases in birth weight (53.28 g), birth length (0.24 cm), and reductions in SGA births (RR 0.94) and stunting (RR 0.82). LNS and MMN groups showed comparable outcome. IFA and MMN were more effective at reducing maternal anemia than LNS.
Goto [33]	Meta-analysis	2019	LNS	LMICs; 58 studies	Lipid-based nutrient supplementation significantly reduced risks of low birthweight, small-for-gestational age, and stunting. It also significantly increased birthweight, birth length, arm circumference, and weight-for-age *z*-score.
Hambidge et al. [59]	RCT	2019	LNS	LMICs (Democratic Republic of the Congo, Guatemala, India, and Pakistan); 7387 females	Starting supplementation ≥3 mo before conception led to higher newborn LAZ scores compared with no supplementation (effect size: +0.19, *P =* 0.0008). Stunting (RR 0.69, *P =* 0.0361), SGA (RR 0.78, *P <* 0.001), and LBW (RR 0.86, *P =* 0.0263) were also reduced compared with no supplementation.
Mekonnen et al. [34]	RCT	2023	Enhancing nutrition and antenatal infection treatment (ENAT)	Ethiopia; 4868 females	The intervention significantly increased mean birth weight by 108 g (from 3044 g in the control to 3152 g in the intervention group, *P =* 0.000) and reduced the incidence of LBW by 36% (4.7% vs. 7.3%, *P =* 0.027) compared with the control group.
Taneja et al. [35]	RCT	2022	Health, nutrition, psychosocial support + WaSH intervention	India; 13,500 females	The intervention package delivered during preconception, pregnancy, and early childhood reduced LBW (IRR 0.76; 95% CI: 0.62, 0.91; ARR −5.59%) and stunting at 24 mo (IRR 0.49; 95% CI: 0.32, 0.75; ARR −7.98%) compared with routine care. Preconception interventions alone reduced LBW (IRR 0.85; 98.3% CI: 0.75, 0.97; ARR −3.80%), whereas pregnancy and early childhood interventions alone significantly reduced stunting at 24 mo (IRR 0.51; 95% CI: 0.38, 0.70; ARR −8.32%).

Abbreviations: ARR, absolute risk reduction; BEP, balanced energy protein; CSB, corn soya blend; CI, confidence interval; GWG, gestational weight gain; HCZ, head circumference z-score; HR, hazard ratio; IFA, iron folic acid; IRR, incidence rate ratio; LAZ, length-for-age z-score; LMICs, low- and middle-income countries; LNS, lipid nutrient supplement; MD, mean difference; MMN, multiple micronutrient supplement; MUAC, mid-upper arm circumference; OR, odds ratio; PLA, participatory learning and action; PTB, preterm birth; RCT, randomized controlled trial; RUSF, ready-to-use supplemental food; WAZ, weight-for-age z-score; WaSH, water, sanitation, and hygiene.

## Current Management Recommendations

International guidance for undernutrition in pregnancy is lacking. WHO guidelines for a positive pregnancy experience recommend that pregnant patients with undernutrition receive nutritional education emphasizing increased intake of energy-dense, locally available foods [36]. As of 2020, WHO supports the use of multiple micronutrient supplements (MMNs) only in the context of limited access to nutritious food, without elucidating these criteria [37]. There are no guidelines for the provision of supplementary foods in terms of type, dose, or duration to pregnant females with undernutrition.

## Types of Intervention

### Educational programs

Educational programs to increase nutrient intake, including provision of recipes for nutrient-dense foods, have shown limited effectiveness in increasing energy and protein intake, improving gestational weight gain, and initiating breastfeeding [18]. Although these programs may reduce risk of preterm birth and low birth weight (LBW) [19,20], their effectiveness depends on food availability, which is often lacking in high-burden settings. In food-insecure environments, specially formulated products that provide nutrients are necessary. The following sections consider such products.

### Adequate micronutrient intake: MMNs

Micronutrient deficiencies are nearly universal among pregnant females in LMICs. MMNs supplements are formulated to deliver standard amounts of 15 common micronutrients as point-of-use supplement packets to pregnant females with inadequate micronutrient intake. The United Nations International Multiple Micronutrient Antenatal Preparation, consisting of vitamins A, B, B2, B3, B6, B12, C, D, and E, copper, folic acid, iodine, iron, selenium, and zinc, is the most commonly utilized [38]. In LMIC settings, MMNs reduce risk of LBW, small-for-gestational age (SGA) infants, and preterm birth [21,22], and may improve long-term child health outcomes including improved body weight, middle upper arm circumference (MUAC), and head circumference [23], and enhanced infant antibody responses to diphtheria–tetanus–pertussis vaccine [24].

The Women’s First Trial in 4 LMICs highlighted the advantages of initiating supplementation preconception or early in pregnancy to optimize maternal and neonatal outcomes [25]. MMNs alone are insufficient to address undernutrition during pregnancy and must be combined with strategies to increase macronutrient intake and reduce inflammation [26].

### Adequate macronutrient intake: BEPs

Macronutrient intake is crucial during pregnancy, with protein and energy needs increasing by 21 g and 420 kcal/d, respectively [39,40]. Protein quality is particularly important, as amino acids are key regulators in growth, development, lactation, and reproduction. Essential amino acids (EAA) cannot be synthesized by the body and must be obtained through diet. Many LMICs rely on plant-based proteins, many of which lack sufficient EAAs, further exacerbating maternal and fetal nutritional deficiencies [41]. Children in LMICs frequently fail to meet EAA requirements even when consuming sufficient total protein due to poor protein quality [42], which likely also applies to pregnant females.

Balanced energy protein supplementations (BEP), which provide ∼25% of energy from protein, are endorsed by WHO for the treatment of undernutrition in pregnancy [36]. The use of BEPs in pregnancy has been shown to decrease the rates of stillbirths, LBW, and SGA infants [19]. BEP products vary widely in terms of composition and administration and include beverages, enriched breads, fortified biscuits, fortified blended flours (FBF), and lipid-based nutrient supplements (LNS) [43]. Most products are locally developed for acceptability among target populations.

FBFs aim to provide 10%–15% of energy from protein, 20%–25% of energy from fat, and approximately two-thirds of daily micronutrient requirements [44]. Originally developed for child undernutrition, FBFs may also be used for undernutrition in pregnancy [45]. Provision of FBF in pregnancy is associated with improvements in maternal anemia [27], reduction in preterm birth rates [27], and improved infant birth anthropometrics [28]. However, FBFs require cooking, have lower energy density than other BEPs, and require higher intake volumes to meet nutritional needs.

LNS are shelf-stable, food-safe products formulated to provide essential nutrients to individuals with undernutrition. LNS typically contains all necessary nutrients, essential fatty acids, lipids, protein, and micronutrients. Although no standard LNS exists for undernutrition in pregnancy, many local programs tailor them to meet specific micronutrient needs during pregnancy [[29], [30], [31],[46], [47], [48]]. LNS come in different sizes: small (SQ-LNS) for prevention, medium (MQ-LNS) for moderate undernutrition, and large (LQ-LNS) for severe undernutrition. MQ-LNS and LQ-LNS are appropriate products for pregnant females with undernutrition. LNS consumption among pregnant females in LMIC settings improves infant weight and length [32], and reduces risk of LBW, SGA, and stunting compared with other supplements [33]; however, these supplements may be less effective among pregnant females with undernutrition. Although there are no standard dosing guidelines, most programs recommend 500–1000 kcal/d for pregnant females with undernutrition.

## Control of Inflammation

Nutritional supplementation alone does not result in adequate improvement to maternal and infant outcomes, as they are less effective in the setting of inflammation and infection. Pregnant females in LMICs have high rates of inflammation [15]. This chronic maternal inflammation is often accompanied by increased metabolic demands and impaired nutrient absorption [13], thereby limiting the effectiveness of food supplements [31,34].

Malaria remains a significant factor leading to poor pregnancy outcomes in endemic areas, representing a complex intersection of infection, inflammation, and malnutrition. Infection triggers inflammatory responses that increase metabolic demands and redirect nutrients toward immune function rather than fetal growth [49]. The inflammatory cytokines produced during malaria directly interfere with iron metabolism and protein synthesis, whereas placental malaria disrupts maternal–fetal nutrient transfer [50]. Conversely, maternal malnutrition increases susceptibility to malaria infection, creating a negative synergy that amplifies both conditions [49]. In such regions, the WHO recommends intermittent preventive treatment in pregnancy with sulfadoxine-pyrimethamine for all pregnant females, regardless of infection status. Test-and-treat strategies—where malaria is only treated if diagnosed—may be considered in areas with lower transmission or concerns about drug resistance [16].

Oral health contributes to systemic inflammation in pregnancy. Poor oral health, particularly periodontal disease and periapical infections, creates a chronic source of bacterial inflammation that elevates circulating inflammatory markers [51]. During pregnancy, when nutritional demands are heightened, this inflammatory burden diverts nutrients away from fetal growth toward inflammatory processes [52]. Periapical infection, a common condition in LMICs, has been associated with adverse pregnancy outcomes, including preterm birth and LBW. Chronic oral infections can trigger systemic inflammatory responses, potentially interfering with the body’s ability to utilize essential nutrients. A study examining associations between maternal dental infections and pregnancy outcomes found that individuals with infections had shorter gestational periods and delivered infants with lower BW and lengths [17]. Additionally, periapical infection was linked to an increased prevalence of stunting, highlighting its potential contribution to early-life growth deficits [17].

Several trials bundling nutritional and other interventions have been performed, though most have not specifically targeted undernourished females [31,34].

Recently, a large RCT in Ethiopia investigated the role of enhanced nutrition and infection [34]. The intervention included screening for asymptomatic bacteriuria, syphilis, anemia, and other maternal infections through point-of-care testing, as well as strengthened antenatal care services—on birth outcomes among pregnant females of all nutritional statuses [34]. The study also introduced nutritional interventions such as MUAC-based malnutrition screening, improved tracking of deworming and iron folic acid supplementation, and enhanced gestational weight monitoring. The intervention demonstrated improved birth weight and a decreased rate of LBW infants among females receiving the intervention [34]. Additionally, a trial in India combined nutrition, psychosocial, and sanitation interventions during pregnancy and demonstrated a reduction in LBW and stunting at 24 mo of life [35]. Notably, these trials have not been targeted at pregnant females with undernutrition. Among pregnant females with undernutrition, a randomized controlled trial in rural Sierra Leone tested a similar hypothesis, combining a novel LNS with routine azithromycin, improved malaria chemoprophylaxis and infection screening compared with standard care and demonstrated improved maternal and infant outcomes, including improved maternal MUAC, increased infant birth weight and length, and infant survival to 6 mo [31].

These limited trials demonstrate the need for additional well-designed trials to determine the optimal nutritional supplement and infection prevention strategies to improve maternal and infant outcomes.

## Ongoing Trials

Several clinical trials aiming to improve the care of pregnant females with undernutrition are ongoing or planned. These include targeted trials of BEP supplements [53,54], trials to enhance nutrition and protein adequacy using locally available foods [55], and trials of novel supplement composition targeted to improve gestation duration and infant cognitive outcomes [56].

## Conclusions and Future Perspectives

Undernutrition in pregnancy remains a substantial problem, especially in LMICs, and has adverse impacts on both the pregnant females and their offspring. Nutritional supplementation alone has not resulted in the desired improvement in outcomes. Management strategies, including both nutrient supplementation and control of inflammation, may ameliorate these impacts.

## Author contributions

The authors’ responsibilities were as follows – DTH, MJM: conceptualized the project; DTH: performed initial literature review and wrote the first draft of the manuscript; AL, KBS, ASK, MJM, DTH: reviewed and edited the manuscript; DTH, MJM: primary responsibility for final content; and all authors: read and approved the final manuscript.

## Declaration of generative AI and AI-assisted technologies in the writing process

During the preparation of this work, the author(s) used no AI technologies.

## Funding

The authors received no funding for the current manuscript.

## Conflict of interest

All authors declare no conflict of interest.
